# Knowledge and awareness of asbestos risk among General Practitioners: Validation of a questionnaire in an area with a high incidence of asbestos-related diseases

**DOI:** 10.1016/j.pmedr.2024.102940

**Published:** 2024-12-05

**Authors:** Marinella Bertolotti, Manuela Tamburro, Angelo Salzo, Antonella Cassinari, Stefania Crivellari, Carlotta Bertolina, Marianna Farotto, Carmen Adesso, Michela Anna Di Palma, Anna Natale, Federico Torregiani, Guglielmo Pacileo, Antonio Maconi, Giancarlo Ripabelli

**Affiliations:** a Research Training Innovation Infrastructure, Research and Innovation Department (DAIRI), Azienda Ospedaliero-Universitaria “S.S. Antonio e Biagio e Cesare Arrigo”, Alessandria, Italy; b Department of Medicine and Health Sciences “Vincenzo Tiberio”, University of Molise, Campobasso, Italy; c Molise Region Health Authority, Italy; d SS. Progetti, Ricerca e Innovazione, “Michele e Pietro Ferrero” Hospital, ASL CN2, Verduno (CN), Italy; e Research and Innovation Department (DAIRI), Azienda Sanitaria Locale, Alessandria, Italy; f School of Specialization in Hygiene and Preventive Medicine, University of Molise, Campobasso, Italy; g General practisioner, Azienda Sanitaria Locale, Alessandria, Italy

**Keywords:** Asbestos exposure, Asbestos-related diseases, General practitioners, Continuing medicine education, Questionnaire, National Priority Contaminated Site

## Abstract

**Objective:**

Given the critical role of general practitioners (GPs) in the early diagnosis and management of asbestos-related diseases (ARDs), and the significant history of asbestos fibres pollution in Alessandria Local Health Authority (ASL AL), this project aimed to assess the knowledge and awareness of asbestos risks, as well as the experience in diagnosing ARDs among GPs working in Alessandria province, Northern Italy.

**Methods:**

A questionnaire was administered to 216 GPs from all ASL AL territorial districts during 26 Territorial Assistance Equipes (EATs) meetings, held from September 2022 to January 2023. It contained 29 questions covering three main areas: ‘knowledge and awareness’, ‘competence and experience’, ‘sociodemographic characteristics and workload’.

**Results:**

Although GPs were aware of the health hazards of asbestos (94 %) and the increased risk of mesothelioma from asbestos exposure (92.6 %), significant disparities and heterogeneity of knowledge were observed among territorial districts and by comparing Casale Monferrato district with all the others, particularly regarding asbestos exposure routes, reporting of occupational diseases, and mesothelioma latency.

**Conclusions:**

This project provides a comprehensive overview of GPs' knowledge, awareness and experience in managing ARDs, providing indications of customised training requirements. This evaluation could be extended to all areas with a history of previous asbestos exposure and provide a useful tool for policy makers to define and plan strategic actions on asbestos. This work could also be adapted to different realities with a history of environmental pollutant exposure other than asbestos, which pose a risk for the development of several diseases.

## Highlights

• Different knowledge among general practisioners on asbestos-related diseases.
• Need to provide specific courses on asbestos diseases to general practisioners.
• Increased knowledge by general practisioners in National Priority Contaminated Sites.
• Limited awareness of familial domestic exposure to asbestos.
• Difficulties in carrying out occupational disease reporting procedures.

## 1 Introduction

The term asbestos refers to a group of naturally occurring fibrous minerals that are used in various industrial processes mainly in the asbestos cement, construction and engineering industries (International Agency for Research on Cancer (IARC), 2012). The health risks associated with asbestos exposure depend on several factors, including the concentration of fibres in the air, the length of exposure, the type and size of the fibres (Pawełczyk and Božek, 2015; Boulanger et al., 2014; Ramada Rodilla et al., 2022). The scientific community has extensively studied the relationship between asbestos exposure and the development of respiratory diseases, including asbestosis, lung cancer, and pleural mesothelioma (Pedley, 1930). In addition, the International Agency for Research on Cancer (IARC) has identified links between asbestos exposure and other types of cancer, which have been considered asbestos-related diseases (ARDs), such as pharyngeal, laryngeal, oesophageal, stomach, colorectal and ovarian cancers (Kwak et al., 2022; Zanardi et al., 2013; Clin et al., 2017; Paris et al., 2017; Nowak et al., 2021; International Agency for Research on Cancer, 2018).

Italy is characterized by 42 National Priority Contaminated Sites (NPCSs) in 19 out of 20 regions, which represent ongoing remediation areas of special environmental interest, acknowledged for its contamination and hazard characteristics due to different environmental matrixes (Araneo et al., 2021).

Alessandria province has a notable history of asbestos fibre pollution, affecting both environmental and occupational settings. Particularly, Casale Monferrato is recognised as asbestos NPCS (https://www.isprambiente.gov.it/en/activities/soil-and-territory/contaminated-sites/contaminated-sites-of-national-interest-sin, 2024), which includes 48 municipalities in Alessandria and surrounding areas (GdL SENTIERI-ReNaM, 2016), and it was the location of the first and largest Italian asbestos-cement manufacturing factory, known as the Eternit company, active from 1907 to 1986 (Comba et al., 2018).

Therefore, due to the presence of major asbestos-producing industries, Casale Monferrato has experienced a high incidence of mesothelioma from occupational exposure (Ferrante et al., 2016). Additionally, many cases of para-occupational exposure (including environmental and family exposure) (Marinaccio et al., 2015) are still reported today.

As a NPCS, Casale Monferrato is characterized by a greater attention for asbestos issue by citizens and territorial associations, and represents a national model in the fight against asbestos, having banned the processing, marketing and use of any containing asbestos fibres five years before the national ban (Natali and de Nardin Budó, 2019).

Despite the ban on asbestos use in Italy since 1992 under Law n. 257/92, ARDs continue to pose a public health challenge in Italy and many other regions worldwide (Barbieri et al., 2020). This is due to the silent clinical progression of ARDs and the associated health and social costs, which also impact insurance, civil and criminal sectors (Musk et al., 2020).

Alessandria Local Health Authority (ASL AL) includes 192 municipalities over an area of 3679 km^2^. It is organized into seven territorial areas and four districts identified as Alessandria and Valenza, Acqui Terme and Ovada, Casale Monferrato, Novi Ligure and Tortona.

In primary care, general practitioners (GPs) know the health status, family, and socio-cultural background, and sometimes the occupational fields of their patients. They are the first point of contact in the healthcare system and play a key role in early disease diagnosis, because they take a patients' extensive anamnesis, examine medical history and assess symptomatology (Quaderni del Ministero della Salute, 2012).

In 2021, a pilot study on asbestos knowledge and awareness using a 29-item questionnaire was carried out among 28 GPs in Molise region, central Italy. The survey revealed a high perception of asbestos risk and good general knowledge among GPs, but there were gaps especially for regulatory procedures (Ripabelli et al., 2018).

According to the Italian National Institute of Health (ISS), the results of this pilot study were considered valuable for planning strategies to improve GPs' awareness of this issue, with the goal of enhancing patient care (Comba and Fazzo, 2017).

Considering these findings, and that asbestos fibre pollution that still characterized the Alessandria area, the Research and Innovation Department (DAIRI) of Alessandria University Hospital carried out a survey aimed to assess the level of knowledge and awareness of asbestos related risks among GPs in ASL AL area, as well as their experience in diagnosing and managing ARDs, throught the administration of a validated questionnaire.

## 2 Methods

### 2.1 Pilot phase of questionnaire validation

A total of 280 GPs work in the Alessandria province. They are members of Territorial Assistance Equipes (EATs) establish in Piedmont region, which include GPs working in similar areas for geographical characteristics and exposure to environmental risk factors with monthly meetings.

The questionnaire, originally used in a previous study (Ripabelli et al., 2018), was revised by expanding the number of questions in the knowledge section.

To validate the updated version, the questionnaire was administered to 17 GPs from both Molise and ASL AL area between June and July 2022. A digital version of the questionnaire was created using the web-based “Research Electronic Data Capture” (REDCap) platform, which complies with current privacy regulations.

GPs were able to access the questionnaire on smartphones, PCs and tablets and it took about 15 min to complete.

All GPs filled in the questionnaire after providing their informed consent.

During this phase, the digital questionnaire was also supplemented with satisfaction questions concerning ease of access, technical issues, clarity of questions and answers, and other improvement suggestions.

### 2.2 Validated questionnaire

The validated questionnaire was presented to GPs in ASL AL during 26 EATs meetings held between September 2022 and January 2023.

The final version of the questionnaire (in supplementary materials) featured 29 questions divided into three sections: the first focused on knowledge of asbestos risks and ARDs consisting of 14 questions; the second on awareness of asbestos risk and ARDs with 8 questions; and the third section on socio-demographic data of GPs, education level and patient caseload, which included 7 questions.

In the knowledge section, the questionnaire contained 25 correct and 36 incorrect answer options.

Each response was scored as follows: 1 point for a correct answer, 0 for no answer and −0.25 for an incorrect answer.

To calculate the overall knowledge level, the formula previously developed was applied (Ripabelli et al., 2018): [number of correct options + (−0.25*number of incorrect options)/25 correct options]*100. Based on the results, knowledge levels were categorized into four quartiles: scarce (0–24 %), sufficient (25–49 %), good (50–74 %) and optimal (75–100 %).

For the awareness section, the total score was calculated by summing the responses to each questions. Furthermore, some questions (Q20, Q21 and Q22) were converted to a Likert scale, and the score was then expressed as a percentage to allow for comparison with the knowledge index. Awareness and competence scores were also divided into four quartiles: inadequate (0–24 %), poor (25–49 %), moderate (50–74 %), and high (75–100 %).

Missing responses (“no answer”) were considered as indicators of no knowledge or competence. Given the targeted group of respondents, hesitation in choosing an answer was interpreted as a potential knowledge gap regarding the specific topic.

To ensure strong participation, the research team attended EAT meetings, explaining the project's objectives and inviting GPs to participate after the read of the privacy data protection and informed consent.

### 2.3 Statistical analysis

Data were analysed in aggregate form. Quantitative data were reported as medians and interquartile ranges (IQR) based on their distribution. Categorical variables were expressed as counts and percentages. To assess statistical significance, Mann-Whitney test was applied for comparisons between two groups while Kruskal-Wallis test was used for comparisons across multiple groups regarding quantitative data. For categorial data, associations were tested using Chi-square test, Fisher's exact test, or Cramer's V. The internal consistency of the Awareness section (Q15-Q19) was assessed using Cronbach's alpha. A two-sided *p*-value <0.05 was considered statistically significant. All statistical analyses were performed using IBM SPSS® version 25 for Windows.

## 3 Results

### 3.1 Study sample

GPs from non-operational EATs at the time of questionnaire distribution and who did not attend EAT meetings, were excluded from the investigation. Hence, 232 out of 280 GPs attended EAT meetings. Of these, only 216 (93,1 %) completed the questionnaire, and 56.5 % were male, with a median age of 60 years (IQR 42.25–64, supplementary table 1).

### 3.2 Knowledge area

Regarding the knowledge section (see Table 1), 59.3 % of GPs had a good level of knowledge and only 18 % an optimal level. The median knowledge score was 63 % (IQR 51–72). There was a statistically significant difference in knowledge levels among the seven districts (*p* = 0.002), and difference became noticeable when comparing Casale Monferrato district to the others (*p* < 0.001). GPs practicing in Casale Monferrato district exhibited a higher median knowledge level of 72 % (IQR 60–78), with 42.6 % achieving an optimal level of knowledge.

**Table 1:** Descriptive statistics related to GPs' overall knowledge indexes in ASL AL districts emerged from the survey filled out during the EAT meetings (September 2022–January 2023).

	TOTAL		ASL AL DISTRICTS														p⁎	Casale Monferrato		Other ASL AL Districts ASL AL		p⁎⁎
			Alessandria		Casale Monferrato		Valenza		Tortona		Novi Ligure		Ovada		Acqui Terme							
	(*n* = 216)		(*n* = 64)		(*n* = 47)		(*n* = 17)		(n = 21)		(*n* = 37)		(*n* = 11)		(*n* = 19)			(n = 47)		(*n* = 169)		
	N	%	N	%	N	%	N	%	N	%	N	%	N	%	N	%		N	%	N	%	
**Knowledge**																						
scarce	5	2.3	2	3.1	0	0.0	0	0	1	4.8	2	5.4	0	0	0	0	0.002	0	0.0	5	3.0	<0.001
sufficient	44	20.4	15	23.4	4	8.5	3	17.6	11	52.4	5	13.5	3	27.3	3	15.8		4	8.5	40	23.7	
good	128	59.3	39	60.9	23	48.9	14	82.4	8	38.0	25	67.6	6	54.5	13	68.4		23	48.9	105	62.1	
optimal	39	18.0	8	12.6	20	42.6	0	0	1	4.8	5	13.5	2	18.2	3	15.8		20	42.6	19	11.2	
Median (IQR)	63 (51–72)		62 (49–71)		72 (60–78)		65 (56–70)		48 (39–63.5)		62 (52.5–69)		64 (47–71)		59 (51–71)			72 (60–78)		61 (49–70)		

⁎ Kruskal-Wallis test.

⁎⁎ Mann-Whitney test.

Regarding the evaluations for the knowledge-related questions, all items assessed showed a statistically significant association with the overall score level (see Table 2). However, a few items did not meet this criteria, including questions about personal protective equipment (Q4f), asbestos-related diseases (Q5b, Q5e), radiological signs of asbestosis (Q9, excluding answers related to pleural plaques), and anatomical structures where mesothelioma does not originate (Q13c). Knowledge evaluations for each ASL AL district are shown in supplementary table 2.

**Table 2:** Knowledge of asbestos risk and ARDs, along with the association of overall scores to corresponding questions.

KNOWLEDGE	TOTAL (n = 216)										Association with knowledge score (p)⁎⁎
	Yes (knowledge)				No (no knowledge)						
	N	%	Overall Index		No		No answer		Overall Index		
			median score	IQR	N	%	N	%	median score	IQR	
Q1) Asbestos is dangerous to human health	203	94.0	64.0	53–73	12	5.5	1	0.5	36.0	22.5–47	<0.001
**Q2) Exposure to asbestos in living or working environments increases the risk of developing mesothelioma**	200	92.6	64.0	54–73	16	7.4	–	–	38.0	25–44	<0.001
**Q3) The following types of asbestos exposure can influence the onset of malignant mesothelioma:**											
a) Occupational exposure	182	84.3	64.5	55.75–73	31	14.3	3	1.4	43.0	30–55	<0.001
b) Family exposure	93	43.1	71.0	64–79	105	48.6	18	8.3	55.0	45–64	<0.001
c) Environmental exposure	147	68.1	66.0	59–74	62	28.7	7	3.2	51.0	39–64	<0.001
Q4) What types of personal protective equipment are effective in reducing the risk of occupational exposure to asbestos?											
a) Disposable coveralls	187	86.6	64.0	53–73	29	13.4	–	–	55.0	36.5–63	0.001
b) Surgical masks	179	82.9	64.0	51–73	37	17.1	–	–	58.0	46–67.5	0.040
c) Filtering face pieces	174	80.6	65.0	57–73.25	42	19.4	–	–	47.0	33.75–55.5	<0.001
d) Washable rubber boots	95	44.0	70.0	59–76	121	56.0	–	–	59.0	47–67	<0.001
e) Hearing protectors	187	86.6	62.0	49–72	29	13.4	–	–	67.0	62–74	0.007
f) Heat resistant clothing	204	94.4	62.0	51–72	12	5.6	–	–	69.5	60.5–72.75	0.182
**Q5) Which of the following diseases are associated with asbestos exposure?**											
a) Pulmonary asbestosis	207	95.8	64.0	52–72	9	4.2	–	–	39.0	29.5–55.5	<0.001
b) Meningioma	209	96.8	63.0	51–72	7	3.2	–	–	59.0	34–67	0.287
c) Pericardial mesothelioma	166	76.9	65.0	55.75–74	50	23.1	–	–	49.5	36.25–62	<0.001
d) Mesothelioma of the tunica vaginalis testis	111	51.4	70.0	62–77	105	48.6	–	–	54.0	44–64	<0.001
e) Non-Hodgkin lymphoma	194	89.8	63.0	51–72.25	22	10.2	–	–	63.5	51.75–67.75	0.657
**Q6) Which of the following are methods of exposure to asbestos?**											
a) Domestic exposure, which refers to those living with someone who has been professionally exposed to asbestos	121	56.0	70.0	62–76.5	95	44.0	–	–	51.0	40–62	<0.001
b) Environmental exposure, which refers to those who live in geographical areas contaminated by asbestos	189	87.5	64.0	54–73	27	12.5	–	–	40.0	30–59	<0.001
c) Occupational exposure, which refers to those who carry out a professional activity in which asbestos is present	206	95.4	64.0	53–72.25	10	4.6	–	–	37.5	22–45.25	<0.001
Q7) Based onthe literature, what is the primary symptom of asbestosis that is useful for directing the diagnosis? c. Exertional dyspnoea, which may progress to dyspnoea at rest	90	41.7	68.5	57.75–77.25	109	50.4	17	7.9	59.5	47–68	<0.001
Q8) When a worker is diagnosed with asbestosis, what does the GP need to report online? a. The physician submits a report online to INAIL	109	50.5	66.0	55.5–76.5	99	45.8	8	3.7	61.0	46–68	<0.001
**Q9) What are the radiological signs of pulmonary asbestosis?**											
a) Pleural plaques	99	45.8	61.0	47–69	117	54.2	–	–	65.0	54.5–74	0.004
b) Fine basal reticular patterns	42	19.4	62.0	52.5–72	174	80.6	–	–	63.5	51–72	0.727
c) A diffuse reticular-nodular pattern	137	63.4	64.0	51–73	79	36.6	–	–	62.0	51–71	0.385
d) Air bronchograms	211	97.7	63.0	51–72	5	2.3	–	–	57.0	22–64.5	0.159
e) Increased intercostal spaces	214	99.1	63.0	51–72	2	0.9	–	–	47.5	n.c.⁎	0.165
Q10) From the literature, what is the average latency period for mesothelioma? e. Over twenty-five years	115	53.2	68.0	58–77	100	46.3	1	0.5	58.0	47–66	<0.001
Q11) Which of the following investigations is most suitable for the diagnosis and staging of pleural mesothelioma? b. Computed Tomography	165	76.4	65.0	55.5–73.5	45	20.8	6	2.8	51.0	40–63	<0.001
Q12) Which statement regarding pleural mesothelioma is correct? d. Asbestos exposure and tobacco smoking are synergistic risk factors	128	59.3	66.5	58.25–74.75	85	39.3	3	1.4	55.0	42.5–65	<0.001
**Q13) From which of the following anatomical structures does mesothelioma NOT originate?**											
a) Lymph nodes	99	45. 8	68.0	58–78	117	54.2	–	–	59.0	45–66.5	<0.001
b) Meninges	112	51.9	65.0	55.25–75	104	48.1	–	–	59.0	48.25–70.75	0.003
c) Pericardium	210	97.2	63.5	51–72	6	2.8	–	–	57.0	41.25–61.25	0.152
d) Myocardium	85	39.4	66.0	55–78	131	60.6	–	–	61.0	49–70	0.002
e) Vaginal tunic	183	84.7	65.0	54–73	33	15.3	–	–	50.0	38.5–58.5	<0.001
**Q14) According to Law 257/92 which regulations relateto the cessation of asbestos use? Asbestos exposure is permitted, with appropriate safety measures for workers, in the following activities** d. Disposal and/or remediation of areas or artefacts containing asbestos	167	77.3	65.0	55–74	41	19.0	8	3.7	50.0	38.5–64	<0.001

⁎ Not calculable because of the low number of responses in the group.

⁎⁎ Mann-Whitney test.

Although there was a high and widespread knowledge about the health hazards of asbestos (94 %) (Table 2, Q1) and the increased risk of mesothelioma due to this exposure (92.6 %, Q2), significant disparities were evident both in the variation of knowledge among different districts and when comparing Casale Monferrato district with the others. The greatest variations in knowledge were particularly noted in the following areas: types of asbestos exposure, procedures for reporting occupational diseases, and understanding of mesothelioma, including latency, diagnostic methods, and risk factors.

Regarding the types of asbestos exposure linked to mesothelioma (Q3), the rates were 84.3 % for occupational exposure 84.3 %, and 68.1 % and 43.1 % for environmental and domestic exposure, respectively. Domestic exposure had the highest rate of missing responses (8.3 %) and knowledge for this type of exposure (Q3b) showed significant differences among the districts (*p* = 0.039), with Casale Monferrato reporting the highest awareness (53.2 %). Statistically significant differences were also observed when comparing Casale Monferrato to the other districts (*p* = 0.008).

Furthermore, when evaluating types of asbestos exposure (Q6), the one least identified by GPs was domestic exposure (Q6a); only 56 % of them recognising it as a mode of exposure, compared to environmental (87.5 %) and occupational exposure (95.4 %). Statistically significant differences in knowledge were found both among the different districts (*p* = 0.003) and between Casale Monferrato and the other districts (*p* = 0.001).

Regarding the symptomatology for asbestosis, only 41.7 % of GPs correctly identified dyspnoea on exertion followed by dyspnoea at rest (Q7). Casale Monferrato had the highest correct response rate (51.1 %). However, there were no statistically significant differences in knowledge among the districts or in comparison to Casale Monferrato.

Regarding the reporting of asbestosis diagnoses for workers exposed to asbestos, only half of the GPs correctly reported the procedure for notifying the National Institute for Insurance against Accidents at Work (INAIL) via electronic platform (Q8). Analysis of the correct and incorrect responses revealed a statistically significant difference between the districts as well as when comparing each district with Casale Monferrato (*p* < 0.001). GPs practising in Casale Monferrato demonstrated better knowledge of the reporting procedure, with 78.7 % answering correctly.

In contrast, on the other hand, when asked about the average latency period for the development of mesothelioma, 46.8 % of GPs failed to provide the correct, which is over 25 years (Q10). There was a statistically significant difference in responses among the districts (p < 0.001), and also when comparing the responses from Casale Monferrato with those from all other districts (*p* = 0.001).

Additionally, the identification of the correct investigation for the diagnosis and staging of pleural mesothelioma (Q11) showed differing levels of knowledge among the districts (*p* = 0.047), as well as in comparison with Casale Monferrato (*p* = 0.048).

Finally, statistically significant differences were found among the districts in correctly identifying the relationship between asbestos exposure and mesothelioma (Q12), specifically that exposure to asbestos and tobacco smoke act as combined risk factors (*p* = 0.004).

### 3.3 Awareness area

The analysis of awareness index (Table 3) revealed a moderate/high overall level of 62 % (median: 54 %, IQR: 46–62). Only 4.6 % of GPs reported a high level of awareness. There were no statistically significant differences in awareness levels when comparing the seven districts or between Casale Monferrato and the other districts (*p* > 0.05).

**Table 3:** Descriptive statistics related to GPs' overall awareness indexes in ASL AL districts emerged from the survey filled out during the EAT meetings (September 2022–January 2023).

	TOTAL		ASL AL DISTRICTS														p⁎	Casale Monferrato		Other ASL AL Districts		p⁎⁎
			Alessandria		Casale Monferrato		Valenza		Tortona		Novi Ligure		Ovada		Acqui Terme							
	(n = 216)		(n = 64)		(n = 47)		(n = 17)		(n = 21)		(n = 37)		(n = 11)		(n = 19)			(n = 47)		(n = 169)		
	N	%	N	%	N	%	N	%	N	%	N	%	N	%	N	%		N	%	N	%	
**Awareness**																	0.065					0.060
inadequate	3	1.4	1	1.6	0	0.0	0	0.0	0	0.0	1	2.7	0	0.0	1	5.3		0	0.0	3	1.8	
poor	79	36.6	30	46.9	14	29.8	4	23.5	5	23.8	14	37.8	4	36.4	8	42.1		14	29.8	65	38.5	
moderate	124	57.4	30	46.9	29	61.7	12	70.6	15	71.4	22	59.5	6	54.5	10	52.6		29	61.7	95	56.2	
high	10	4.6	3	4.6	4	8.5	1	5.9	1	4.8	0	0.0	1	9.1	0	0.0		4	8.5	6	3.5	
Median (IQR)	54 (46–62)		51 (43–59.5)		57 (49–65)		57 (50–64)		62 (50–68)		52 (45.5–60.5)		57 (48–69)		52 (45–60)			57 (49–65)		52 (45.5–62)		

⁎ Kruskal-Wallis test.

⁎⁎ Mann-Whitney test.

The reliability of the scale for questions Q15 to Q19 (12 items) was more than acceptable, with a Cronbach's alpha of 0.73.

Regarding the awareness items (Table 4), all associations between the overall score and the individual areas were statistically significant. Awareness evaluations for each ASL AL district are reported in supplementary table 3.

**Table 4:** Awareness/competence on asbestos risk and ARDs and association of overall score with the corresponding questions.

AWARENESS	TOTAL (n = 216)										Association with awareness score (p)⁎
	Yes (aware)				No (not aware)						
			Overall Index		No		No answer		Overall Index		
	N	%	median score	IQR	N	%	N	%	median score	IQR	
*Q15) Which of the following actions related to occupational diseases fall within your scope of responsibility?*											
a) Diagnosis	175	81.0	57.0	48–63	35	16.2	6	2.8	43.0	34.5–52	<0.001
b) Complaint/ formal notification	140	64.8	57.0	49–65	66	30.6	10	4.6	49.0	40.50–57	<0.001
c) Compilation of medical certificates	142	65.7	57.0	49–65	57	26.4	17	7.9	48.5	41.5–57	<0.001
*What factors contribute to the difficulty in reporting an occupational disease?*											
a) Inexperience with bureaucratic procedures	17	7.9	72.0	62.5–80.5	184	85.2	15	6.9	52.0	46–60	<0.001
b) Challenges in carrying out bureaucratic procedures	12	5.6	71.0	62.25–77.75	191	88.4	13	6.0	52.0	46–60	<0.001
c) Lack of understanding of diagnostic criteria	76	35.2	62.5	57–71	117	54.2	23	10.6	49.0	43–57	<0.001
d) Lack of time	86	39.8	60.0	52–68.25	105	48.6	25	11.6	50.0	43–58	<0.001
e) Inadequate professional updating (ECM, etc.)	53	24.5	63.0	57.5–72	147	68.1	16	7.4	51.0	43–58	<0.001
f) Complexity of the List of occupational diseases for which reporting is mandatory	29	13.4	68.0	61–73	170	78.7	17	7.9	52.0	45–58	<0.001
Q17) Do you feel that your current level of scientific and professional knowledge on asbestos-related diseases is sufficient to address patient inquiries regarding occupational diseases and workplace accidents?”	55	25.5	58.0	49–71	160	74.0	1	0.5	52.0	46–60	<0.001
Q18) Do you find the Continuing Medical Education (CME) in the Piedmont Region to be adequate in covering asbestos-related diseases?	38	17.6	59.0	48.75–72	177	81.9	1	0.5	52.0	46–62	0.004
Q19) Are you well-informed about the occupational activities of your patients?	136	63.0	57.0	49–65	79	36.5	1	0.5	49.0	42–57.75	<0.001
Q20) In the last 12 months, have you followed Continuing Medical Education courses (ECM) that covered or included topics on asbestos-related diseases?	12	5.6	61.5	57–73.25	203	93.9	1	0.5	52.0	46–62	0.010
Q21) In the past 12 months, have you seen or treated patients with asbestos-related conditions?	65	30.1	58.0	49–68	151	69.9	–	–	52. 0	45–60	0.001
Q22) In the last 5 years, how many occupational disease reports related to asbestos exposure have you submitted?	53	24.5	57.0	51–70	161	74.6	2	0.9	52.0	45–60	0.002

⁎ Mann-Whitney test.

Regarding GPs' awareness, statistically significant differences were found among the districts for the following issues: competence in occupational diseases, especially on the diagnosis and lack of knowledge of the diagnostic criteria for recognising ARDs; complexity of the list of occupational diseases; inadequate and unsatisfactory level of scientific and professional training on ARDs; examining patients with ARDs and the compilation of occupational disease reports.

About GPs' competence in diagnosing occupational diseases (Q15a), no significant differences were observed between districts, except for a difference with Casale Monferrato (*p* = 0.042).

In terms of barriers for reporting occupational diseases, statistically significant differences were found for the lack of knowledge of diagnostic criteria (Q16c), both among districts (*p* < 0.001) and compared to Casale Monferrato (*p* = 0.001). Differences were also observed for lack of time (Q16d) and the complexity of the occupational diseases (Q16f) among districts (*p* = 0.036 and *p* = 0.049 respectively), but not when compared to Casale Monferrato. Conversely, this difference was evident for the item on inadequate professional updating, but not among districts (p = 0.036).

A key finding from the analysis is that many GPs (74 % with a score ≤ 3) did not feel that their current level of scientific and professional training on ARDs is sufficient to answer patients' questions on occupational diseases and workplace accidents (Q17). This was statistically significant both among districts and compared to Casale Monferrato (*p* ≤0.001). Only 8.5 % of GPs in Casale Monferrato rated their training as inadequate, compared to higher rates in other districts.

Furthermore, 81.9 % of GPs considered as inadequate the quality of Continuing Medical Education (CME) on asbestos and ARDs (Q18) and only 5.6 % reported attending CME courses in the past 12 months that included topics on ARDs (Q20).

Additionally, 69.9 % of GPs (1.4 % who failed to recall) reported they had not evaluated ARD cases in the previous year (Q21). Significant differences were observed between districts and in comparison with Casale Monferrato. Notably, 78.7 % of GPs in Casale Monferrato, reported their experience in visiting patients with ARD (*p* < 0.001). The median number of patients visited was 2.50 (IQR 2–6.25) in Casale Monferrato, compared to 1 (IQR 1–2) in the other districts (p < 0.001).

Approximately 75 % of GPs had no experience in filling occupational disease reports in the last 5 years (Q22), while 5.1 % had submitted at least five. Conversely, 38.3 % of GPs in Casale Monferrato district submitted reports during the period (14.9 % of whom had completed at least five certificates), compared to 20.7 % in the other districts (*p* = 0.005).

Finally, from the analysis of demographic and workload data of GPs (supplementary table 4) an increase in knowledge was observed in younger GPs (p < 0.001) and thus lower seniority (*p* = 0.006).

GPs with a General Medicine qualification demonstrated a higher overall knowledge index, compared to those with a specialization (*p* = 0.001). Furthermore, the overall knowledge index was higher in GPs who had visited and treated patients with ARDs in the past year, compared to those who had not (p < 0.001).

Concerning workload activity, knowledge was higher in those with a higher patients' daily contact (*p* = 0.029) and daily outpatient hour (*p* = 0.037).

## 4 Discussion

GPs play a crucial role in the early diagnosis and correct management of patients with ARDs. For this reason, this project aimed to explore the levels of knowledge, awareness and experience of ARDs among those GPs practising in ASL AL. The assessment focused on clinical features, prevention, patient communication, legal and administrative procedures, also assessing differences between Casale Monferrato district, with its history of asbestos exposure and serious health consequences on citizens' health, and the others.

This is the first project to comprehensively evaluate the level of knowledge and competence regarding asbestos risk, as well as the experience of GPs in diagnosing and managing ARDs in ASL AL. This initiative is the second of its kind in Italy, following a pilot project organized by the University of Molise in 2021 in its regional territory.

ASL AL covers an extensive area, but the asbestos issue is particularly focused in Casale Monferrato. The characteristics of this district, recognised as a NPCS, contribute to the higher level of knowledge observed among GPs working there. As a national role model in the fight against asbestos, GPs in Casale Monferrato possess greater awareness and understanding of this issue.

The analysis of knowledge and awareness/competence indexes highlights critical areas requiring attention, particularly the factors that most impacted overall scores.

The level of knowledge was very high on the danger of asbestos for health and the increased risk of developing mesothelioma that it causes, as well as on the role of occupational exposure in their occurrence, while the determinant of mesothelioma due to exposure in the family home setting was the less recognised.

Based on the differences in knowledge observed among GPs in Casale Monferrato with respect to all the other ASL AL districts, the analysis reveal the need for more in-depth knowledge on ARDs, even in view of the unknown asbestos exposures emerging with the new mesothelioma cases and those still occurring even outside the NPCS of Casale Monferrato.

These results suggest that GPs' education on environmental pollutant-related disease should be implemented not only in NPCSs and should start since the university, including scientific knowledge, differential diagnostics and medical-legal aspects.

However, the asbestos issue is not limited to Italy, but it represents a global concern. Although banned in many countries, there are still several realities in which asbestos is still used, and others in which only recently has been banned (http://www.asbestosnation.org/facts/asbestos-bans-around-the-world/, 2024; Burki, 2024), with an increasing amount of diagnosis of illness related to asbestos exposure (Marsili et al., 2016; Chen et al., 2022).

Indeed, there are globally developed research data which have claimed that asbestos, besides contributing to several types of cancer, thus representing a serious public health risk, is a problem especially in developing countries and in those where asbestos use bans and coordinated management plans have not been implemented (Thives et al., 2022).

Moreover, the Global Cancer Observatory, Cancer Incidence in Five Continents Plus, and Global Burden of Disease were recently consulted for mesothelioma incidence and its risk factors worldwide. Although a substantial decrease in mesothelioma trends has been observed, especially in highly developed countries, probably attributable to the complete ban several years ago on asbestos use, stable and increasing trends have been observed in several countries (Huang et al., 2023).

In this context Casale Monferrato represents a model in the fight against asbestos, especially due to the epidemiological studies conducted since 1980s (Magnani et al., 1993; Magnani and Leporati, 1998; Magnani et al., 1995; Magnani et al., 1987; Botta et al., 1991; Magnani et al., 2023), hence this experience can also support countries where this problem is emerging. In these realities, information should be provided on the knowledge of risk related to asbestos exposure, as well as on the disease features (such as latency and clinical signs for correct differential diagnosis) to promptly start the medical-legal recognition process, where it exists, through an efficient educational program to healthcare professionals and physicians.

Furthermore, it is imperative to mention the heterogeneity of NPCSs. Beyond the well-known asbestos pollution, many other NPCSs in Italy are responsible for the development of several diseases. The most notable pollutants include chemical plants, coal mines, petrochemical plants and refineries, steel industries, harbors areas, landfills of hazardous or mixed waste, electric power plants and first-generation incinerators (Zona et al., 2023).

Because of the heterogeneity in the contamination of NPCSs, this survey could be adapted to other territorial areas with different environmental contamination, as well as in neighboring areas. The questionnaire used could be a valuable research tool for homogeneous data collection among different NPCSs, useful for describing potential requirements.

## 5 Conclusions

This survey highlights the need to provide for GPs topic-specific update courses that would enable them to increase knowledge on recognising ARDs symptoms, and to guide the patient towards early diagnosis, likewise in the occupational disease notification process. It would be useful to extend this assessment to all areas with a history of previous asbestos exposure, including NPCSs and those areas that despite showing this issue do ot have such recognition.

Furthermore, the developed questionnaire could be a useful tool for decision makers for defining and planning strategic actions on asbestos. For the same purpose, it could be adapted to different realities also with a history of environmental pollutants exposure different from asbestos.

## Funding

This research received no specific grant from any funding agency in the public, commercial, or not-for-profit sectors.

## CRediT authorship contribution statement

**Marinella Bertolotti:** Writing – review & editing, Writing – original draft, Supervision, Project administration, Methodology, Investigation, Data curation, Conceptualization. **Manuela Tamburro:** Writing – review & editing, Methodology. **Angelo Salzo:** Writing – review & editing, Methodology. **Antonella Cassinari:** Writing – review & editing, Writing – original draft, Project administration, Methodology, Data curation. **Stefania Crivellari:** Writing – review & editing, Writing – original draft, Project administration, Investigation. **Carlotta Bertolina:** Writing – review & editing, Writing – original draft, Project administration, Investigation. **Marianna Farotto:** Writing – review & editing, Project administration, Investigation. **Carmen Adesso:** Writing – review & editing, Methodology. **Michela Anna Di Palma:** Writing – review & editing, Methodology. **Anna Natale:** Writing – review & editing, Methodology. **Federico Torregiani:** Writing – review & editing, Project administration, Conceptualization. **Guglielmo Pacileo:** Writing – review & editing, Project administration, Conceptualization. **Antonio Maconi:** Writing – review & editing, Supervision, Conceptualization. **Giancarlo Ripabelli:** Writing – review & editing, Methodology.

## Declaration of competing interest

The authors declare that they have no known competing financial interests or personal relationships that could have appeared to influence the work reported in this paper.

## Data Availability

Data will be made available on request.

## Appendix A Supplementary data

Supplementary material 1 Image 1

Supplementary material 2 Image 2

Supplementary material 3 Image 3

Supplementary material 4 Image 4

Supplementary material 5 Image 5

## References

[bb0005] Araneo F., Bartolucci E., Vecchio A. (2021).

[bb0010] Barbieri P.G., Calisti R., Silvestri S., Calabresi C., Consonni D., Angelini A., Carnevale F., Cavariani F., Sala O. (2020 Jan-Feb). A proposito dell’amianto e del Position Paper della Società italiana di medicina del lavoro sull’amianto (About the asbestos and the Position Paper on asbestos of the Italian Society of Occupational Medicine). Epidemiol. Prev..

[bb0015] Botta M., Magnani C., Terracini B., Bertolone G.P., Castagneto B., Cocito V., DeGiovanni D., Paglieri P. (1991). Mortality from respiratory and digestive cancers among asbestos cement workers in Italy. Cancer Detect. Prev..

[bb0020] Boulanger G., Andujar P., Pairon J.C. (2014). Quantification of short and long asbestos fibers to assess asbestos exposure: a review of fiber size toxicity. Environ. Health.

[bb0025] Burki T. (2024 May). EPA ruling to ban chrysotile asbestos. Lancet Oncol..

[bb0030] Chen J., Wang C., Zhang J., Zhang T., Liang H., Mao S., Li H., Wang Z. (2022 Nov 3). A comparative study of the disease burden attributable to asbestos in Brazil, China, Kazakhstan, and Russia between 1990 and 2019. BMC Public Health.

[bb0035] Clin B., Thaon I., Boulanger M., Brochard P., Chamming’s S., Gislard A., Lacourt A., Luc A., Ogier G., Paris C., Pairon J.C. (2017 Nov). Cancer of the esophagus and asbestos exposure. Am. J. Ind. Med..

[bb0040] Comba P., Fazzo L. (2017). Rapporti ISTISAN 17/37.

[bb0045] Comba P., D’Angelo M., Fazzo L., Magnani C., Marinaccio A., Mirabelli D., Terracini B. (2018 Apr-Jun). Mesothelioma in Italy: the Casale Monferrato model to a national epidemiological surveillance system. Ann. Ist. Super. Sanita.

[bb0050] Ferrante D., Mirabelli D., Tunesi S., Terracini B., Magnani C. (2016 Mar). Pleural mesothelioma and occupational and non-occupational asbestos exposure: a case-control study with quantitative risk assessment. Occup. Environ. Med..

[bb0055] GdL SENTIERI-ReNaM (2016). SENTIERI - studio epidemiologico nazionale dei territori e degli insediamenti esposti a rischio da inquinamento: l’incidenza del mesotelioma (SENTIERI - Epidemiological study of residents in national priority contaminated sites: incidence of mesothelioma). Epidemiol. Prev..

[bb0060] (2024). http://www.asbestosnation.org/facts/asbestos-bans-around-the-world/.

[bb0065] (2024). https://www.isprambiente.gov.it/en/activities/soil-and-territory/contaminated-sites/contaminated-sites-of-national-interest-sin.

[bb0070] Huang J., Chan S.C., Pang W.S., Chow S.H., Lok V., Zhang L., Lin X., Lucero-Prisno D.E., Xu W., Zheng Z.J., Elcarte E., Withers M., Wong M.C.S. (2023 Jun). NCD global health research group, Association of Pacific rim Universities (APRU). Global incidence, risk factors, and temporal trends of mesothelioma: a population-based study. J. Thorac. Oncol..

[bb0075] International Agency for Research on Cancer (2018). Asbestos (chrysotile, amosite, crocidolite, tremolire, actinolite, and anthophyllite) 100C. https://monographs.iarc.who.int.

[bb0080] International Agency for Research on Cancer (IARC) (2012).

[bb0085] Kwak K., Kang D., Paek D. (2022 Mar). Environmental exposure to asbestos and the risk of lung cancer: a systematic review and meta-analysis. Occup. Environ. Med..

[bb0090] Magnani C., Leporati M. (1998 Feb). Mortality from lung cancer and population risk attributable to asbestos in an asbestos cement manufacturing town in Italy. Occup. Environ. Med..

[bb0095] Magnani C., Terracini B., Bertolone G.P., Castagneto B., Cocito V., De Giovanni D., Paglieri P., Botta M. (1987). Mortalità per tumori e altre malattie del sistema respiratorio tra i lavoratori del cemento-amianto a Casale Monferrato. Uno studio di coorte storico [Mortality from tumors and other diseases of the respiratory system in cement-asbestos workers in Casale Monferrato. A historical cohort study]. Med Lav.

[bb0100] Magnani C., Terracini B., Ivaldi C., Botta M., Budel P., Mancini A., Zanetti R. (1993 Sep). A cohort study on mortality among wives of workers in the asbestos cement industry in Casale Monferrato, Italy. Br. J. Ind. Med..

[bb0105] Magnani C., Terracini B., Ivaldi C., Botta M., Mancini A., Andrion A. (1995 Jun). Pleural malignant mesothelioma and non-occupational exposure to asbestos in Casale Monferrato, Italy. Occup. Environ. Med..

[bb0110] Magnani C., Mensi C., Binazzi A., Marsili D., Grosso F., Ramos-Bonilla J.P., Ferrante D., Migliore E., Mirabelli D., Terracini B., Consonni D., Degiovanni D., Lia M., Cely-García M.F., Giraldo M., Lysaniuk B., Comba P., Marinaccio A. (2023 Jan 4). The Italian experience in the development of mesothelioma registries: a pathway for other countries to address the negative legacy of Asbestos. Int. J. Environ. Res. Public Health.

[bb0115] Marinaccio A., Binazzi A., Bonafede M., Corfiati M., Di Marzio D., Scarselli A., Verardo M., Mirabelli D., Gennaro V., Mensi C., Schallemberg G., Merler E., Negro C., Romanelli A., Chellini E., Silvestri S., Cocchioni M., Pascucci C., Stracci F., Ascoli V., Trafficante L., Angelillo I., Musti M., Cavone D., Cauzillo G., Tallarigo F., Tumino R., Melis M., ReNaM Working Group (2015 Sep). Malignant mesothelioma due to non-occupational asbestos exposure from the Italian national surveillance system (ReNaM): epidemiology and public health issues. Occup. Environ. Med..

[bb0120] Marsili D., Terracini B., Santana V.S., Ramos-Bonilla J.P., Pasetto R., Mazzeo A., Loomis D., Comba P., Algranti E. (2016 May 12). Prevention of Asbestos-related disease in countries currently using Asbestos. Int. J. Environ. Res. Public Health.

[bb0125] Musk A.W., de Klerk N., Reid A., Hui J., Franklin P., Brims F. (2020 Jun 1). Asbestos-related diseases. Int. J. Tuberc. Lung Dis..

[bb0130] Natali L., de Nardin Budó M. (2019). A sensory and visual approach for comprehending environmental victimization by the asbestos industry in Casale Monferrato. Eur. J. Criminol..

[bb0135] Nowak D., Schmalfeldt B., Tannapfel A., Mahner S. (2021 May). Asbestos exposure and ovarian cancer - a gynaecological occupational disease. Background, mandatory notification, practical approach. Geburtshilfe Frauenheilkd..

[bb0140] Paris C., Thaon I., Hérin F., Clin B., Lacourt A., Luc A., Coureau G., Brochard P., Chamming’s S., Gislard A., Galan P., Hercberg S., Wild P., Pairon J.C., Andujar P. (2017 Mar). Occupational Asbestos exposure and incidence of Colon and Rectal cancers in French men: the Asbestos-related diseases cohort (ARDCo-nut). Environ. Health Perspect..

[bb0145] Pawełczyk A., Božek F. (2015 Jul). Health risk associated with airborne asbestos. Environ. Monit. Assess..

[bb0150] Pedley F.G. (1930 Feb). ASBESTOSIS. Can. Med. Assoc. J..

[bb0155] Quaderni del Ministero della Salute (2012). Stato dell’arte e prospettive in materia di contrasto alle patologie asbesto-correlate.

[bb0160] Ramada Rodilla J.M., Calvo Cerrada B., Serra Pujadas C., Delclos G.L., Benavides F.G. (2022). Fiber burden and asbestos-related diseases: an umbrella review. Gac. Sanit..

[bb0165] Ripabelli G., Tamburro M., Di Tella D., Carrozza F., Sammarco M.L. (2018 Feb). Asbestos exposures, mesothelioma incidence and mortality, and awareness by general practitioners in the Molise region, Central Italy. J. Occup. Environ. Med..

[bb0170] Thives L.P., Ghisi E., Thives Júnior J.J., Vieira A.S. (2022 Oct 1). Is asbestos still a problem in the world? A current review. J. Environ. Manag..

[bb0175] Zanardi F., Salvarani R., Cooke R.M., Pirastu R., Baccini M., Christiani D., Curti S., Risi A., Barbieri A., Barbieri G., Mattioli S., Violante F.S. (2013 Jan). Carcinoma of the pharynx and tonsils in an occupational cohort of asphalt workers. Epidemiology.

[bb0180] Zona A., Fazzo L., Benedetti M., Bruno C., Vecchi S., Pasetto R., Minichilli F., De Santis M., Nannavecchia A.M., Di Fonzo D., Contiero P., Ricci P., Bisceglia L., Manno V., Minelli G., Santoro M., Gorini F., Ancona C., Scondotto S., Soggiu M.E., Scaini F., Beccaloni E., Marsili D., Villa M.F., Maifredi G., Magoni M., Iavarone I., Gruppo di lavoro SENTIERI 2019-2022 (2023). SENTIERI - Studio epidemiologico nazionale dei territori e degli insediamenti esposti a rischio da inquinamento. Sesto Rapporto [SENTIERI - Epidemiological Study of Residents in National Priority Contaminated Sites Sixth Report].

