# Supplementary material for: Knowledge and awareness of asbestos risk among General Practitioners: Validation of a questionnaire in an area with a high incidence of asbestos-related diseases
Source: Prev Med Rep. 2024 Dec 5;49:102940. doi: 10.1016/j.pmedr.2024.102940 (PMC11697718; doi:10.1016/j.pmedr.2024.102940)
Supplement: Supplementary file 1 — Supplementary material 1 [file mmc1.docx]

Supplementary Table 1: GPs demographic and professional characteristics and organization of their outpatient activity (7 questions).

|  | **TOTAL** | | **DISTRICT ASL AL** | | | | | | | | | | | | | | p |
| --- | --- | --- | --- | --- | --- | --- | --- | --- | --- | --- | --- | --- | --- | --- | --- | --- | --- |
|  |  |  | **Alessandria** | | **Casale Monferrato** | | **Valenza** | | **Tortona** | | **Novi Ligure** | | **Ovada** | | **Acqui Terme** | |  |
|  | **(n=216)** | | **(n=64)** | | **(n=47)** | | **(n=17)** | | **(n=21)** | | **(n=37)** | | **(n=11)** | | **(n=19)** | |  |
|  | N | % | N | % | N | % | N | % | N | % | N | % | N | % | N | % |  |
| **Gender** |  |  |  |  |  |  |  |  |  |  |  |  |  |  |  |  | 0.037^*^ |
| Male | 122 | 56.5 | 25 | 39.1 | 31 | 66.0 | 12 | 70.6 | 13 | 61.9 | 23 | 62.2 | 5 | 45.5 | 13 | 68.4 |  |
| Female | 94 | 43.5 | 39 | 60.9 | 16 | 34.0 | 5 | 29.4 | 8 | 38.1 | 14 | 37.8 | 6 | 54.5 | 6 | 31.6 |  |
| **Age (years)** |  |  |  |  |  |  |  |  |  |  |  |  |  |  |  |  |  |
| <45 | 60 | 27.8 | 20 | 31.3 | 15 | 31.9 | 3 | 17.6 | 4 | 19.1 | 10 | 27.0 | 7 | 63.6 | 1 | 5.3 |  |
| 45-60 | 53 | 24.5 | 21 | 32.8 | 7 | 14.9 | 5 | 29.4 | 7 | 33.3 | 6 | 16.2 | 0 | 0.0 | 7 | 36.8 |  |
| >60 | 103 | 47.7 | 23 | 35.9 | 25 | 53.2 | 9 | 52.9 | 10 | 47.6 | 21 | 56.8 | 4 | 36.4 | 11 | 57.9 |  |
| Median (IQR) | 60 (42.25-64) | | 56.5 (40-64) | | 61 (35-64) | | 61 (56.5-64.5) | | 59 (48-67) | | 62 (44-63.5) | | 39 (32-65) | | 63 (57-67) | | 0.088^**^ |
| Range | 27-70 | | 31-69 | | 29-69 | | 32-66 | | 32-69 | | 31-69 | | 27-69 | | 41-70 | |  |
| **Medical specialization** |  |  |  |  |  |  |  |  |  |  |  |  |  |  |  |  | 0.197^*^ |
| GPs Course | 69 | 31.9 | 25 | 39.1 | 16 | 34.0 | 4 | 23.5 | 4 | 19.1 | 14 | 37.8 | 4 | 36.4 | 2 | 10.5 |  |
| Specialization | 74 | 34.3 | 17 | 26.6 | 14 | 29.8 | 7 | 41.2 | 12 | 57.1 | 14 | 37.8 | 4 | 36.4 | 6 | 31.6 |  |
| NoGPs Course or specialization | 73 | 33.8 | 22 | 34.3 | 17 | 36.2 | 6 | 35.3 | 5 | 23.8 | 9 | 24.4 | 3 | 27.2 | 11 | 57.9 |  |
| **Working experience** |  |  |  |  |  |  |  |  |  |  |  |  |  |  |  |  |  |
| ≤10 years | 48 | 22.2 | 16 | 25.0 | 16 | 34.1 | 2 | 11.8 | 3 | 14.3 | 7 | 18.9 | 4 | 36.3 | 0 | 0.0 |  |
| 11-29 | 55 | 25.5 | 23 | 35.9 | 5 | 10.6 | 3 | 17.7 | 7 | 33.3 | 7 | 18.9 | 3 | 27.3 | 7 | 36.8 |  |
| 30-35 | 68 | 31.5 | 18 | 28.2 | 15 | 31.9 | 9 | 52.9 | 4 | 19.1 | 16 | 43.3 | 2 | 18.2 | 4 | 21.1 |  |
| >35 | 45 | 20.8 | 7 | 10.9 | 11 | 23.4 | 3 | 17.6 | 7 | 33.3 | 7 | 18.9 | 2 | 18.2 | 8 | 42.1 |  |
| Median(IQR) | 30 (12-35) | | 26.5 (10.5-34) | | 30 (7-35) | | 31 (26.5-32.5) | | 30 (19-38) | | 31 (14-35) | | 12 (6-34) | | 31 (26-41) | | 0.098^**^ |

^*^ Chi-square test; ^**^ Kruskal-Wallis test.
