# Supplementary material for: Knowledge and awareness of asbestos risk among General Practitioners: Validation of a questionnaire in an area with a high incidence of asbestos-related diseases
Source: Prev Med Rep. 2024 Dec 5;49:102940. doi: 10.1016/j.pmedr.2024.102940 (PMC11697718; doi:10.1016/j.pmedr.2024.102940)
Supplement: Supplementary file 2 — Supplementary material 2 [file mmc2.docx]

Supplementary Table 2: knowledge on asbestos risk and ARDs for each ASL AL district

| **KNOWLEDGE** | **DISTRICT ASL AL** | | | | | | | **p*^**^*** | **Casale Monferrato compared**  **to others** | | **p*^***^*** |
| --- | --- | --- | --- | --- | --- | --- | --- | --- | --- | --- | --- |
|  | **Alessandria (n=64)** | **Casale Monferrato (n=47)** | **Valenza**  **(n=17)** | **Tortona**  **(n=21)** | **Novi Ligure**  **(n=37)** | **Ovada**  **(n=11)** | **Acqui Terme (n=19)** |  | **Casale Monferrato (n=47)** | **Other District ASL AL**  **(n=169)** |  |
|  | **N (%)** | **N (%)** | **N (%)** | **N (%)** | **N (%)** | **N (%)** | **N (%)** |  | **N (%)** | **N (%)** |  |
| **Q1)** | Yes 61 (95.3)  No 3 (4.7) | Yes 46 (97.9)  No 1 (2.1) | Yes 17 (100.0)  No 0 (0.0) | Yes 17 (81.0)  No 4 (19.0) **n.a. 1 (4.8)* | Yes 35 (94.6)  No 2 (5.4) | Yes 10 (90.9)  No 1 (9.1) | Yes 17 (89.5)  No 2 (10.5) | 0.363 | Yes 46 (97.9)  No 1 (2.1) | Yes 157 (92.9)  No 12 (7.1) | 0.243 |
| **Q2)** | Yes 61 (95.3)  No 3 (4.7) | Yes 44 (93.6)  No 3 (6.4) | Yes 16 (94.1)  No 1 (5.9) | Yes 17 (81.0)  No 4 (19.0) | Yes 34 (91.9)  No 3 (8.1) | Yes 10 (90.9)  No 1 (9.1) | Yes 18 (94.7)  No 1 (5.3) | 0.475 | Yes 44 (93.6)  No 3 (6.4) | Yes 156 (92.3)  No 13 (7.7) | 0.749 |
| **Q3) a** |  |  |  |  |  |  |  |  |  |  |  |
|  | Yes 53 (82.8)  No 11 (17.2) | Yes 43 (91.5)  No 4 (8.5) | Yes 16 (94.1)  No 1 (5.9) | Yes 15 (71.4)  No 6 (28.6)  **n.a. 1 (4.8)* | Yes 28 (75.7)  No 9 (24.3)  **n.a. 2 (5.4)* | Yes 10 (90.9)  No 1 (9.1) | Yes 17 (89.5)  No 2 (10.5) | 0.408 | Yes 43 (91.5)  No 4 (8.5) | Yes 139 (82.2)  No 30 (17.8) | 0.163 |
| **Q3) b** | Yes 27 (42.2)  No 37 (57.8)  **n.a. 9 (14.1)* | Yes 25 (53.2)  No 22 (46.8)  **n.a. 1 (2.1)* | Yes 8 (47.1)  No 9 (52.9) | Yes 5 (23.8)  No 16 (76.2)  **n.a. 2 (9.5)* | Yes 16 (43.2)  No 21 (56.8)  **n.a. 3 (8.1)* | Yes 5 (45.5)  No 6 (54.5)  **n.a. 1 (9.1)* | Yes 7 (36.8)  No 12 (63.2)  **n.a. 2 (10.5)* | 0.039 | Yes 25 (53.2)  No 22 (46.8) | Yes 68 (40.2)  No 101 (59.8) | 0.008 |
| **Q3) c** | Yes 44 (68.8)  No 20 (31.2)  **n.a. 4 (6.3)* | Yes 35 (74.5)  No 12 (25.5) | Yes 12 (70.6)  No 5 (29.4) | Yes 10 (47.6)  No 11 (52.4)  **n.a. 1 (4.8)* | Yes 23 (62.2)  No 14 (37.8)  **n.a. 1 (2.7)* | Yes 9 (81.8)  No 2 (18.2) | Yes 14 (73.7)  No 5 (26.3)  **n.a. 1 (5.3)* | 0.331 | Yes 35 (74.5)  No 12 (25.5) | Yes 112 (66.3)  No 57 (33.7) | 0.336 |
| **Q4) a** |  |  |  |  |  |  |  |  |  |  |  |
|  | Yes 56 (87.5)  No 8 (12.5) | Yes 43 (91.5)  No 4 (8.5) | Yes 13 (76.5)  No 4 (23.5) | Yes 19 (90.5)  No 2 (9.5) | Yes 32 (86.5)  No 5 (13.5) | Yes 10 (90.9)  No 1 (9.1) | Yes 14 (73.7)  No 5 (26.3) | 0.459 | Yes 43 (91.5)  No 4 (8.5) | Yes 144 (85.2)  No 25 (14.8) | 0.264 |
| Q4) b | Yes 50 (78.1)  No 14 (21.9) | Yes 41 (87.2)  No 6 (12.8) | Yes 13 (76.5)  No 4 (23.5) | Yes 16 (76.2)  No 5 (23.8) | Yes 33 (89.2)  No 4 (10.8) | Yes 10 (90.9)  No 1 (9.1) | Yes 16 (84.2)  No 3 (15.8) | 0.628 | Yes 41 (87.2)  No 6 (12.8) | Yes 138 (81.7)  No 31 (18.3) | 0.369 |
| Q4) c | Yes 50 (78.1)  No 14 (21.9) | Yes 40 (85.1)  No 7 (14.9) | Yes 15 (88.2)  No 2 (11.8) | Yes 16 (76.2)  No 5 (23.8) | Yes 29 (78.4)  No 8 (21.6) | Yes 8 (72.7)  No 3 (27.3) | Yes 16 (84.2)  No 3 (15.8) | 0.873 | Yes 40 (85.1)  No 7 (14.9) | Yes 134 (79.3)  No 35 (20.7) | 0.373 |
| Q4) d | Yes 30 (46.9)  No 34 (53.1) | Yes 19 (40.4)  No 28 (59.6) | Yes 8 (47.1)  No 9 (52.9) | Yes 9 (42.9)  No 12 (57.1) | Yes 13 (35.1)  No 24 (64.9) | Yes 5 (45.5)  No 6 (54.5) | Yes 11 (57.9)  No 8 (42.1) | 0.782 | Yes 19 (40.4)  No 28 (59.6) | Yes 76 (45.0)  No 93 (55.0) | 0.579 |
| Q4) e | Yes 52 (81.3)  No 12 (18.7) | Yes 39 (83.0)  No 8 (17.0) | Yes 16 (94.1)  No 1 (5.9) | Yes 20 (95.2)  No 1 (4.8) | Yes 32 (86.5)  No 5 (13.5) | Yes 9 (81.8)  No 2 (18.2) | Yes 19 (100.0)  No 0 (0.0) | 0.283 | Yes 39 (83.0)  No 8 (17.0) | Yes 148 (87.6)  No 21 (12.4) | 0.414 |
| Q4) f | Yes 59 (92.2)  No 5 (7.8) | Yes 44 (93.6)  No 3 (6.4) | Yes 17 (100.0)  No 0 (0.0) | Yes 20 (95.2)  No 1 (4.8) | Yes 34 (91.9)  No 3 (8.1) | Yes 11 (100.0)  No 0 (0.0) | Yes 19 (100.0)  No 0 (0.0) | 0.686 | Yes 44 (93.6)  No 3 (6.4) | Yes 160 (94.7)  No 9 (5.3) | 0.726 |
| **Q5) a** |  |  |  |  |  |  |  |  |  |  |  |
|  | Yes 61 (95.3)  No 3 (4.7) | Yes 46 (97.9)  No 1 (2.1) | Yes 16 (94.1)  No 1 (5.9) | Yes 19 (90.5)  No 2 (9.5) | Yes 36 (97.3)  No 1 (2.7) | Yes 10 (90.9)  No 1 (9.1) | Yes 19 (100.0)  No 0 (0.0) | 0.696 | Yes 46 (97.9)  No 1 (2.1) | Yes 161 (95.3)  No 8 (4.7) | 0.687 |
| Q5) b | Yes 60 (93.8)  No 4 (6.3) | Yes 47 (100.0)  No 0 (0.0) | Yes 17 (100.0)  No 0 (0.0) | Yes 21 (100.0)  No 0 (0.0) | Yes 35 (94.6)  No 2 (5.4) | Yes 10 (90.9)  No 1 (9.1) | Yes 19 (100.0)  No 0 (0.0) | 0.313 | Yes 47 (100.0)  No 0 (0.0) | Yes 162 (95.9)  No 7 (4.1) | 0.351 |
| Q5) c | Yes 45 (70.3)  No 19 (29.7) | Yes 38 (80.9)  No 9 (19.1) | Yes 13 (76.5)  No 4 (23.5) | Yes 16 (76.2)  No 5 (23.8) | Yes 29 (78.4)  No 8 (21.6) | Yes 9 (81.8)  No 2 (18.2) | Yes 16 (84.2)  No 3 (15.8) | 0.840 | Yes 38 (80.9)  No 9 (19.1) | Yes 128 (75.7)  No 41 (24.3) | 0.462 |
| Q5) d | Yes 35 (54.7)  No 29 (45.3) | Yes 24 (51.1)  No 23 (48.9) | Yes 8 (47.1)  No 9 (52.9) | Yes 11 (52.4)  No 10 (47.6) | Yes 18 (48.6)  No 19 (51.4) | Yes 6 (54.5)  No 5 (45.5) | Yes 9 (47.4)  No 10 (52.6) | 0.995 | Yes 24 (51.1)  No 23 (48.9) | Yes 87 (51.5)  No 82 (48.5) | 0.960 |
| Q5) e | Yes 56 (87.5)  No 8 (12.5) | Yes 46 (97.9)  No 1 (2.1) | Yes 14 (82.4)  No 3 (17.6) | Yes 20 (95.2)  No 1 (4.8) | Yes 33 (89.2)  No 4 (10.8) | Yes 10 (90.9)  No 1 (9.1) | Yes 15 (78.9)  No 4 (21.1) | 0.245 | Yes 46 (97.9)  No 1 (2.1) | Yes 148 (87.6)  No 21 (12.4) | 0.053 |
| **Q6) a** |  |  |  |  |  |  |  |  |  |  |  |
|  | Yes 34 (53.1)  No 30 (46.9) | Yes 36 (76.6)  No 11 (23.4) | Yes 10 (58.8)  No 7 (41.2) | Yes 6 (28.6)  No 15 (71.4) | Yes 18 (48.6)  No 19 (51.4) | Yes 9 (81.8)  No 2 (18.2) | Yes 8 (42.1)  No 11 (57.9) | 0.003 | Yes 36 (76.6)  No 11 (23.4) | Yes 85 (50.3)  No 84 (49.7) | 0.001 |
| Q6) b | Yes 55 (85.9)  No 9 (14.1) | Yes 45 (95.7)  No 2 (4.3) | Yes 15 (88.2)  No 2 (11.8) | Yes 16 (76.2)  No 5 (23.8) | Yes 32 (86.5)  No 5 (13.5) | Yes 9 (81.8)  No 2 (18.2) | Yes 17 (89.5)  No 2 (10.5) | 0.428 | Yes 45 (95.7)  No 2 (4.3) | Yes 144 (82.2)  No 25 (14.8) | 0.053 |
| Q6) c | Yes 61 (95.3)  No 3 (4.7) | Yes 44 (93.6)  No 3 (6.4) | Yes 17 (100.0)  No 0 (0.0) | Yes 19 (90.5)  No 2 (9.5) | Yes 35 (94.6)  No 2 (5.4) | Yes 11 (100.0)  No 0 (0.0) | Yes 19 (100.0)  No 0 (0.0) | 0.704 | Yes 44 (93.6)  No 3 (6.4) | Yes 162 (95.9)  No 7 (4.1) | 0.456 |
| **Q7)** | Yes 28 (43.8)  No 36 (56.2)  **n.a. 2 (3.1)* | Yes 24 (51.1)  No 23 (48.9) **n.a. 5 (10.6)* | Yes 3 (17.6)  No 14 (82.4) **n.a. 7 (41.2)* | Yes 7 (33.3)  No 14 (66.7) **n.a. 1 (4.8)* | Yes 14 (37.8)  No 23 (62.2) | Yes 5 (45.5)  No 6 (54.5) | Yes 9 (47.4)  No 10 (52.6) **n.a. 2 (10.5)* | 0.321 | Yes 24 (51.1)  No 23 (48.9) | Yes 66 (39.1)  No 103 (60.9) | 0.140 |
| **Q8)** | Yes 32 (50.0)  No 32 (50.0)  **n.a. 3 (4.7)* | Yes 37 (78.7)  No 10 (21.3) | Yes 4 (23.5)  No 13 (76.5) **n.a. 2 (11.8)* | Yes 4 (19.0)  No 17 (81.0) | Yes 16 (43.2)  No 21 (56.8) **n.a. 3 (8.1)* | Yes 6 (54.5)  No 5 (45.5) | Yes 10 (56.2)  No 9 (47.4) | <0.001 | Yes 37 (78.7)  No 10 (21.3) | Yes 72 (42.6)  No 97 (57.4) | <0.001 |
| **Q9) a** |  |  |  |  |  |  |  |  |  |  |  |
|  | Yes 28 (43.8)  No 36 (56.2) | Yes 16 (34.0)  No 31 (66.0) | Yes 6 (35.3)  No 11 (64.7) | Yes 14 (66.7)  No 7 (33.3) | Yes 19 (51.4)  No 18 (48.6) | Yes 6 (54.5)  No 5 (45.5) | Yes 10 (52.6)  No 9 (47.4) | 0.216 | Yes 16 (34.0)  No 31 (66.0) | Yes 83 (49.1)  No 86 (50.9 | 0.067 |
| Q9) b | Yes 13 (20.3)  No 51 (79.7) | Yes 8 (17.0)  No 39 (83.0) | Yes 1 (5.9)  No 16 (94.1) | Yes 5 (23.8)  No 16 (76.2) | Yes 7 (18.9)  No 30 (81.1) | Yes 3 (27.3)  No 8 (72.7) | Yes 5 (26.3)  No 14 (73.7) | 0.748 | Yes 8 (17.0)  No 39 (83.0) | Yes 34 (20.1)  No 135 (79.9) | 0.635 |
| Q9) c | Yes 34 (53.1)  No 30 (46.9) | Yes 30 (63.8)  No 17 (36.2) | Yes 10 (58.8)  No 7 (41.2) | Yes 16 (76.2)  No 5 (23.8) | Yes 26 (70.3)  No 11 (29.7) | Yes 6 (54.5)  No 5 (45.5) | Yes 15 (78.9)  No 4 (21.1) | 0.264 | Yes 30 (63.8)  No 17 (36.2) | Yes 107 (63.3)  No 62 (36.7) | 0.948 |
| Q9) d | Yes 61 (95.3)  No 3 (4.7) | Yes 47 (100.0)  No 0 (0.0) | Yes 17 (100.0)  No 0 (0.0) | Yes 20 (95.2)  No 1 (4.8) | Yes 37 (100.0)  No 0 (0.0) | Yes 10 (90.9)  No 1 (9.1) | Yes 19 (100.0)  No 0 (0.0) | 0.300 | Yes 47 (100.0)  No 0 (0.0) | Yes 164 (97.0)  No 5 (3.0) | 0.588 |
| Q9) e | Yes 64 (100.0)  No 0 (0.0) | Yes 47 (100.0)  No 0 (0.0) | Yes 17 (100.0)  No 0 (0.0) | Yes 20 (95.2)  No 1 (4.8) | Yes 36 (97.3)  No 1 (2.7) | Yes 11 (100.0)  No 0 (0.0) | Yes 19 (100.0)  No 0 (0.0) | 0.410 | Yes 47 (100.0)  No 0 (0.0) | Yes 167 (98.8)  No 2 (1.2) | >0.999 |
| **Q10)** | Yes 30 (46.9)  No 34 (53.1) | Yes 35 (74.5)  No 12 (25.5) | Yes 16 (94.1)  No 1 (5.9) | Yes 6 (28.6)  No 15 (71.4) | Yes 17 (45.9)  No 20 (54.1) | Yes 7 (63.6)  No 4 (36.4) **n.a. 1 (9.1)* | Yes 4 (21.1)  No 15 (78.9) | <0.001 | Yes 35 (74.5)  No 12 (25.5) | Yes 80 (47.3)  No 89 (52.7) | 0.001 |
| **Q11)** | Yes 47 (73.4)  No 17 (26.6) | Yes 41 (87.2)  No 6 (12.8) **n.a. 2 (4.3)* | Yes 12 (70.6)  No 5 (29.4) **n.a. 3 (17.6)* | Yes 14 (66.7)  No 7 (33.3) | Yes 33 (89.2)  No 4 (10.8) | Yes 7 (63.6)  No 4 (36.4) **n.a. 1 (9.1)* | Yes 11 (57.9)  No 8 (42.1) | 0.047 | Yes 41 (87.2)  No 6 (12.8) | Yes 124 (73.4)  No 45 (26.6) | 0.048 |
| **Q12)** | Yes 38 (59.4)  No 26 (40.6) | Yes 28 (59.6)  No 19 (40.4) | Yes 9 (52.9)  No 8 (47.1) **n.a. 3 (17.6)* | Yes 9 (42.9)  No 12 (57.1) | Yes 26 (70.3)  No 11 (29.7) | Yes 7 (63.6)  No 4 (36.4) | Yes 11 (57.9)  No 8 (42.1) | 0.004 | Yes 28 (59.6)  No 19 (40.4) | Yes 100 (59.2)  No 69 (40.8) | 0.960 |
| **Q13) a** |  |  |  |  |  |  |  |  |  |  |  |
|  | Yes 28 (43.8)  No 36 (56.2) | Yes 24 (51.1)  No 23 (48.9) | Yes 8 (47.1)  No 9 (52.9) | Yes 4 (19.0)  No 17 (81.0) | Yes 19 (51.4)  No 18 (48.6) | Yes 6 (54.5)  No 5 (45.5) | Yes 10 (52.6)  No 9 (47.4) | 0.249 | Yes 24 (51.1)  No 23 (48.9) | Yes 75 (44.4)  No 94 (55.6) | 0.416 |
| Q13) b | Yes 30 (46.9)  No 34 (53.1) | Yes 26 (55.3)  No 21 (44.7) | Yes 11 (64.7)  No 6 (35.3) | Yes 9 (42.9)  No 12 (57.1) | Yes 19 (51.4)  No 18 (48.6) | Yes 5 (45.5)  No 6 (54.5) | Yes 12 (63.2)  No 7 (36.8) | 0.701 | Yes 26 (55.3)  No 21 (44.7) | Yes 86 (50.9)  No 83 (49.1) | 0.591 |
| Q13) c | Yes 62 (96.9) No 2 (3.1) | Yes 47 (100.0)  No 0 (0.0) | Yes 16 (94.1)  No 1 (5.9) | Yes 20 (95.2)  No 1 (4.8) | Yes 36 (97.3)  No 1 (2.7) | Yes 10 (90.9)  No 1 (9.1) | Yes 19 (100.0)  No 0 (0.0) | 0.616 | Yes 47 (100.0)  No 0 (0.0) | Yes 163 (96.4)  No 6 (3.6) | 0.344 |
| Q13) d | Yes 21 (32.8)  No 43 (67.2) | Yes 23 (48.9)  No 24 (51.1) | Yes 6 (35.3)  No 11 (64.7) | Yes 10 (47.6)  No 11 (52.4) | Yes 14 (37.8)  No 23 (62.2) | Yes 4 (36.4)  No 7 (63.6) | Yes 7 (36.8)  No 12 (63.2) | 0.704 | Yes 23 (48.9)  No 24 (51.1) | Yes 62 (36.7)  No 107 (63.3) | 0.128 |
| Q13) e | Yes 53 (82.8)  No 11 (17.2) | Yes 42 (89.4)  No 5 (10.6) | Yes 15 (88.2)  No 2 (11.8) | Yes 13 (61.9)  No 8 (38.1) | Yes 33 (89.2)  No 4 (10.8) | Yes 10 (90.9)  No 1 (9.1) | Yes 17 (89.5)  No 2 (10.5) | 0.095 | Yes 42 (89.4)  No 5 (10.6) | Yes 141 (83.4)  No 28 (16.6) | 0.318 |
| **Q14)** | Yes 50 (78.1)  No 14 (21.9)  **n.a. 1 (1.6)* | Yes 40 (85.1)  No 7 (14.9) **n.a. 1 (2.1)* | Yes 12 (70.6)  No 5 (29.4) **n.a. 3 (17.6)* | Yes 15 (71.4)  No 6 (28.6) | Yes 30 (81.1)  No 7 (18.9) | Yes 7 (63.6)  No 4 (36.4) **n.a. 1 (9.1)* | Yes 13 (68.4)  No 6 (31.6) **n.a. 2 (10.5)* | 0.565 | Yes 40 (85.1)  No 7 (14.9) | Yes 127 (75.1)  No 42 (24.9) | 0.149 |

*^*^the weight of “no answers”in determining wrong answers is reported in notes*

*^**^ Chi-square test; ^***^ Fisher's exact test.*
