# Supplementary material for: Knowledge and awareness of asbestos risk among General Practitioners: Validation of a questionnaire in an area with a high incidence of asbestos-related diseases
Source: Prev Med Rep. 2024 Dec 5;49:102940. doi: 10.1016/j.pmedr.2024.102940 (PMC11697718; doi:10.1016/j.pmedr.2024.102940)
Supplement: Supplementary file 3 — Supplementary material 3 [file mmc3.docx]

Supplementary Table 3: Awareness on asbestos risk and ARDs for each ASL AL district.

| **AWARENESS** | **DISTRICT ASL AL** | | | | | | |  | **Casale Monferrato compared**  **to others** | |  |
| --- | --- | --- | --- | --- | --- | --- | --- | --- | --- | --- | --- |
|  | **Alessandria (n=64)** | **Casale Monferrato (n=47)** | **Valenza (n=17)** | **Tortona (n=21)** | **Novi Ligure (n=37)** | **Ovada (n=11)** | **Acqui Terme (n=19)** |  | **Casale Monferrato (n=47)** | **Other District ASL AL (n=169)** |  |
|  | **N (%)** | **N (%)** | **N (%)** | **N (%)** | **N (%)** | **N (%)** | **N (%)** | **p*^**^*** | **N (%)** | **N (%)** | **p*^***^*** |
| **Q15) a** |  |  |  |  |  |  |  |  |  |  |  |
|  | Yes 47 (37.4)  No 17 (26.6)  **n.a. 3 (4.7)* | Yes 44 (93.6)  No 3 (6.4) | Yes 14 (82.4)  No 3 (17.6) **n.a. 1 (5.9)* | Yes 16 (76.2)  No 5 (23.8) **n.a. 1 (4.8)* | Yes 29 (78.4)  No 8 (21.6) | Yes 10 (90.9)  No 1 (9.1) | Yes 15 (78.9)  No 4 (21.1) **n.a. 1 (5.3)* | 0.387 | Yes 44 (93.6)  No 3 (6.4) | Yes 131 (77.5)  No 38 (22.5) | 0.042 |
| **Q15) b** | Yes 40 (62.5)  No 24 (37.5)  **n.a. 5 (7.8)* | Yes 24 (51.1)  No 23 (48.9) **n.a. 1 (2.1)* | Yes 13 (76.5)  No 4 (23.5) | Yes 17 (81.0)  No 4 (19.0) **n.a. 1 (4.8)* | Yes 25 (67.6)  No 12 (32.4) **n.a. 2 (5.4)* | Yes 9 (81.8)  No 2 (18.2) | Yes 12 (63.2)  No 7 (36.8) **n.a. 1 (5.3)* | 0.356 | Yes 24 (51.1)  No 23 (48.9) | Yes 116 (68.6)  No 53 (31.4) | 0.110 |
| **Q15) c** | Yes 45 (70.3)  No 19 (29.7)  **n.a. 6 (9.4)* | Yes 29 (61.7)  No 18 (38.3) **n.a. 1 (2.1)* | Yes 11 (64.7)  No 6 (35.3) | Yes 16 (76.2)  No 5 (23.8) **n.a. 2 (9.5)* | Yes 22 (59.5)  No 15 (40.5) **n.a. 4 (10.8)* | Yes 8 (72.7)  No 3 (27.3) | Yes 11 (57.9)  No 8 (42.1) **n.a. 4 (21.1)* | 0.663 | Yes 29 (61.7)  No 18 (38.3) | Yes 113 (66.9)  No 56 (33.1) | 0.564 |
| **Q16) a** |  |  |  |  |  |  |  |  |  |  | 0.307 |
|  | Yes 6 (9.4)  No 58 (90.6)  **n.a. 6 (9.4)* | Yes 1 (2.1)  No 46 (97.9) **n.a. 4 (8.5)* | Yes 3 (17.6)  No 14 (82.4) **n.a. 1 (5.9)* | Yes 2 (9.5)  No 19 (90.5) | Yes 2 (5.4)  No 35 (94.6) **n.a. 2 (5.4)* | Yes 1 (9.1)  No 10 (90.9) | Yes 2 (10.5)  No 17 (89.5) **n.a. 2 (10.5)* | 0.848 | Yes 1 (2.1)  No 46 (97.9) | Yes 16 (9.5)  No 153 (90.5) |  |
| **Q16) b** | Yes 6 (9.4)  No 58 (90.6) **n.a. 4 (6.3)* | Yes 2 (4.3)  No 45 (95.7) **n.a. 2 (4.3)* | Yes 0 (0.0)  No 17 (100.0) | Yes 3 (14.3)  No 18 (85.7) | Yes 0 (0.0)  No 37 (100.0) **n.a. 5 (13.5)* | Yes 0 (0.0)  No 11 (100.0) | Yes 1 (5.3)  No 18 (94.7)  **n.a. 2 (10.5)* | 0.334 | Yes 2 (4.3)  No 45 (95.7) | Yes 10 (5.9)  No 159 (94.1) | 0.556 |
| **Q16) c** | Yes 13 (20.3)  No 51 (79.7) **n.a. 8 (12.5)* | Yes 25 (53.2)  No 22 (46.8) **n.a. 6 (12.8)* | Yes 10 (58.8)  No 7 (41.2) **n.a. 1 (5.9)* | Yes 8 (38.1)  No 13 (61.9) | Yes 11 (29.7)  No 26 (70.3) **n.a. 6 (16.2)* | Yes 4 (36.4)  No 7 (63.6) | Yes 5 (26.3)  No 14 (73.7) **n.a. 2 (10.5)* | <0.001 | Yes 25 (53.2)  No 22 (46.8) | Yes 51 (30.2)  No 118 (69.8) | 0.001 |
| **Q16) d** | Yes 24 (37.5)  No 40 (62.5)  **n.a. 9 (14.1)* | Yes 11 (23.4)  No 36 (76.6) **n.a. 7 (14.9)* | Yes 8 (47.1)  No 9 (52.9) **n.a. 1 (5.9)* | Yes 14 (66.7)  No 7 (33.3) **n.a. 1 (4.8)* | Yes 19 (51.4)  No 18 (48.6) **n.a. 5 (13.5)* | Yes 4 (36.4)  No 7 (63.6) | Yes 6 (31.6)  No 13 (68.4) **n.a. 2 (10.5)* | 0.036 | Yes 11 (23.4)  No 36 (76.6) **n.a. 7 (14.9)* | Yes 75 (44.4)  No 94 (55.6) | 0.058 |
| **Q16) e** | Yes 18 (28.1)  No 46 (71.9) **n.a. 6 (9.4)* | Yes 16 (34.0)  No 31 (66.0) **n.a. 4 (8.5)* | Yes 3 (17.6)  No 14 (82.4) | Yes 6 (28.6)  No 15 (71.4) | Yes 5 (13.5)  No 32 (86.5) **n.a. 4 (10.8)* | Yes 2 (18.2)  No 9 (81.8) | Yes 3 (15.8)  No 16 (84.2) **n.a. 2 (10.5)* | 0.392 | Yes 16 (34.0)  No 31 (66.0) | Yes 37 (21.9)  No 132 (78.1) | 0.036 |
| **Q16) f** | Yes 11 (17.2)  No 53 (82.8) **n.a. 7 (10.9)* | Yes 2 (4.3)  No 45 (95.7) **n.a. 3(6.4)* | Yes 2 (11.8)  No 15 (88.2) **n.a. 1 (5.9)* | Yes 5 (23.8)  No 16 (76.2) **n.a. 2 (9.5)* | Yes 4 (10.8)  No 33 (89.2) **n.a. 2 (5.4)* | Yes 1 (9.1)  No 10 (90.9) | Yes 4 (21.1)  No 15 (78.9) **n.a. 2 (10.5* | 0.049 | Yes 2 (4.3)  No 45 (95.7) | Yes 27 (16.0)  No 142 (84.0) | 0.097 |
| **Q17** | Yes 14 (21.9)  No 50 (78.1) | Yes 22 (46.8)  No 25 (53.2) | Yes 5 (29.4)  No 12 (70.6) | Yes 2 (9.5)  No 19 (90.5) | Yes 7 (18.9)  No 30 (81.1) **n.a. 1 (2.7)* | Yes 1 (9.1)  No 10 (90.9) | Yes 4 (21.1)  No 15 (78.9) | <0.001 | Yes 22 (46.8)  No 25 (53.2) | Yes 33 (19.5)  No 136 (80.5) | <0.001 |
| **Q18)** | Yes 11 (17.2)  No 53 (82.8)  **n.a. 1 (1.6)* | Yes 8 (17.0)  No 39 (83.0) | Yes 1 (5.9)  No 16 (94.1) | Yes 3 (14.3)  No 18 (85.7) | Yes 8 (21.6)  No 29 (78.4) | Yes 2 (18.2)  No 9 (81.8) | Yes 5 (26.3)  No 14 (73.7) | 0.360 | Yes 8 (17.0)  No 39 (83.0) | Yes 30 (17.8)  No 139 (82.2) | 0.163 |
| **Q19)** | Yes 37 (57.8)  No 27 (42.2) | Yes 32 (68.1)  No 15 (31.9) | Yes 11 (64.7)  No 6 (35.3) | Yes 14 (66.7)  No 7 (33.3) | Yes 22 (59.5)  No 15 (40.5) **n.a. 1 (2.7)* | Yes 8 (72.7)  No 3 (27.3) | Yes 12 (63.2)  No 7 (36.8) | 0.557 | Yes 32 (68.1)  No 15 (31.9) | Yes 104 (61.5)  No 65 (38.5) | 0.413 |
| **Q20** | Yes 3 (4.7)  No 61 (95.3) | Yes 3 (6.4)  No 44 (93.6) | Yes 0 (0.0)  No 17 (100.0) | Yes 3 (14.3)  No 18 (85.7) | Yes 2 (5.4)  No 35 (94.6) **n.a. 1 (2.8)* | Yes 0 (0.0)  No 11 (100.0) | Yes 1 (5.3)  No 18 (94.7) | 0.562 | Yes 3 (6.4)  No 44 (93.6) | Yes 9 (5.3)  No 160 (94.7) | 0.726 |
| **Q21)** | Yes 13 (20.3)  No 51 (79.7) | Yes 37 (78.7)  No 10 (21.3) | Yes 5 (29.4)  No 12 (70.6) | Yes 2 (9.5)  No 19 (90.5) | Yes 4 (10.8)  No 33 (89.2) | Yes 3 (27.3)  No 8 (72.7) | Yes 1 (5.3)  No 18 (94.7) | <0.001 | Yes 37 (78.7)  No 10 (21.3) | Yes 28 (16.6)  No 141 (83.4) | <0.001 |
| **Q22)** | Yes 11 (17.2)  No 53 (82.8) | Yes 18 (38.3)  No 29 (61.7) | Yes 6 (35.3)  No 11 (64.7) **n.a. 1 (5.9)* | Yes 6 (28.6)  No 15 (71.4) | Yes 5 (13.5)  No 32 (86.5) **n.a. 1 (2.7)* | Yes 2 (18.2)  No 9 (81.8) | Yes 5 (26.3)  No 14 (73.7) | 0.103 | Yes 18 (38.3)  No 29 (61.7) | Yes 35 (20.7)  No 134 (79.3) | 0.005 |

*^*^the weight of “no answer” in determining wrong answers is reported in notes*

*^**^ Chi-square test; ^***^ Fisher's exact test*
