# Supplementary material for: Knowledge and awareness of asbestos risk among General Practitioners: Validation of a questionnaire in an area with a high incidence of asbestos-related diseases
Source: Prev Med Rep. 2024 Dec 5;49:102940. doi: 10.1016/j.pmedr.2024.102940 (PMC11697718; doi:10.1016/j.pmedr.2024.102940)
Supplement: Supplementary file 4 — Supplementary material 4 [file mmc4.docx]

Supplementary Table 4: Knowledge indexes related to GPs demographic and professional characteristics.

| **Knowledge** | **DEMOGRAPHIC AND SERVICE CHARACTERISTICS** | | | | | | | | | | | | **PATIENT RELATIONSHIP CHARACTERISTICS** | | | | | | | | |
| --- | --- | --- | --- | --- | --- | --- | --- | --- | --- | --- | --- | --- | --- | --- | --- | --- | --- | --- | --- | --- | --- |
|  | **Age**  (n=216) | | | p^*^ | **GP’s Qualification**  (n=143) | | p^**^ | **Seniority** (n=216) | | | | p^*^ | **Intensity of commitment (contacts/day)**  (n=216) | | p^**^ | **Intensity of commitment (hours/day)**  (n=216) | | p^**^ | **Visited ARD patients (last year)**  (n=216) | | p^**^ |
|  | <45  years old | 45 – 60  years old | >60  years old |  | General Medicine Training Course | Specialization |  | ≤10 years in service | 11 - 29 years in service | 30 - 35 years in service | >35 years in service |  | <50 | ≥50 |  | ≤20 | >20 |  | Yes | No/doesn’t remember |  |
|  | (n=60) | (n=53) | (n=103) |  | (n=69) | (n=74) |  | (n=48) | (n=55) | (n=68) | (n=45) |  | (n=87) | (n=129) |  | (n=83) | (n=133) |  | (n=65) | (n=151) |  |
|  | N (%) | N (%) | N (%) |  | N (%) | N (%) |  | N (%) | N (%) | N (%) | N (%) |  | N (%) | N (%) |  | N (%) | N (%) |  | N (%) | N (%) |  |
| scarce | 0 (0.0) | 0 (0.0) | 5 (4.9) |  | 0 (0.0) | 3 (4.1) |  | 0 (0.0) | 1 (1.8) | 2 (2.9) | 2 (4.4) |  | 2 (2.3) | 3 (2.3) |  | 4 (4.8) | 1 (0.8) |  | 0 (0.0) | 5 (3.4) |  |
| sufficient | 5 (8.3) | 14 (26.4) | 25 (24.3) |  | 7 (10.1) | 21 (28.4) |  | 3 (6.2) | 13 (23.6) | 17 (25.0) | 11 (24.4) |  | 21 (24.1) | 23 (17.8) |  | 20 (24.1) | 24 (18.0) |  | 8 (12.3) | 36 (23.8) |  |
| good | 39 (65.0) | 31 (58.5) | 58 (56.3) |  | 45 (65.3) | 41 (55.3) |  | 33 (68.8) | 27 (49.1) | 43 (63.3) | 25 (55.6) |  | 54 (62.1) | 74 (57.4) |  | 49 (59.1) | 79 (59.4) |  | 33 (50.8) | 95 (62.9) |  |
| optimal | 16 (26.7) | 8 (15.1) | 15 (14.5) |  | 17 (24.6) | 9 (12.2) |  | 12 (25.0) | 14 (25.5) | 6 (8.8) | 7 (15.6) |  | 10 (11.5) | 29 (22.5) |  | 10 (12.0) | 29 (21.8) |  | 24 (36.9) | 15 (9.9) |  |
| Median (IQR) | 67  (61.25-75) | 62  (49-71.5) | 59  (47-70) | <0.001 | 67  (58.5-74.5) | 60.5  (47-69) | 0.001 | 67  (61.0–74.5) | 64  (49.0-75.0) | 60  (48.25-69.75) | 60  (47.5-71.5) | 0.006 | 59  (48-70) | 64  (53-73.5) | 0.029 | 60  (46-71) | 64  (52.5-73.5) | 0.037 | 71  (61.5-78) | 60  (49-69) | <0.001 |

^*^ Kruskal-Wallis test; ^**^ Mann-Whitney test
