# Supplementary material for: Knowledge and awareness of asbestos risk among General Practitioners: Validation of a questionnaire in an area with a high incidence of asbestos-related diseases
Source: Prev Med Rep. 2024 Dec 5;49:102940. doi: 10.1016/j.pmedr.2024.102940 (PMC11697718; doi:10.1016/j.pmedr.2024.102940)
Supplement: Supplementary file 5 — Supplementary material 5 [file mmc5.docx]

**Questionario sulla valutazione della conoscenza e consapevolezza del rischio da amianto nei Medici di Medicina Generale, in un territorio ad alta incidenza di patologie asbesto-correlate**

Le chiediamo cortesemente di partecipare ad un'indagine sulla percezione e la conoscenza delle malattie asbesto correlate da parte dei Medici di Medicina Generale dell'ASL AL. La ricerca è condotta dalla S.S. Epidemiologia afferente alla S.C. Infrastruttura Ricerca Formazione ed Innovazione (IRFI) - Dipartimento Interaziendale Attività Integrate Ricerca e Innovazione (DAIRI) A.O./ASL AL, in collaborazione con il Dipartimento di Medicina e Scienze della Salute V. Tiberio dell’Università degli Studi del Molise di Campobasso.

La partecipazione è su base volontaria pertanto potrà interrompere la collaborazione in qualsiasi momento, senza dover fornire le motivazioni di tale intenzione.

**Informativa privacy e trattamento dati**

Il Titolare del trattamento è l’A.O.AL, con sede legale in Via Venezia, 16, 15121 Alessandria che tratterà i Suoi dati personali in forma anonima ed aggregata per finalità di ricerca. Lo strumento di cui si avvale la racco1ta dati è REDCap (Research Electronic Data Capturo), applicazione web conforme alla normativa in materia di Studi clinici GCP E6 (R2) — IHC e Privacy (Regolamento Europeo 2016/679 — GDPR).

□ ACCETTO □ NON ACCETTO

***AMBITO DELLA CONOSCENZA***

***Le seguenti domande sono volte a rilevare le conoscenze sulle malattie legate all'amianto.

Su una scala da 1 a 5, dove 1 è “Completamente in disaccordo” e 5 è “Completamente d'accordo”, indichi la sua opinione per ognuna delle affermazioni che seguiranno.***

**1.** “L’asbesto è pericoloso per la salute umana”.

1□□□□□5

**2.** “L’esposizione ad asbesto negli ambienti di vita o di lavoro aumenta il rischio di sviluppare il mesotelioma”.

1□□□□□5

3. Le seguenti modalità di esposizione all’asbesto possono influenzare l’insorgenza del mesotelioma maligno:

- a) Esposizione ***professionale***  1□□□□□5

- b) Esposizione ***familiare*** 1□□□□□5

- c) Esposizione ***ambientale***  1□□□□□5

4. Quali tra questi sono dispositivi di protezione individuale utili ad evitare il rischio di esposizione professionale all’asbesto? (È possibile barrare più di una risposta)

a. Tute integrali monouso

b. Mascherina chirurgica

c. Facciali filtranti

d. Stivali in gomma lavabili

e. Otoprotettori

f. Indumenti anticalore

5. Quali delle seguenti patologie sono correlate all’esposizione ad amianto? (È possibile barrare più di una risposta)

a. Asbestosi polmonare

b. Meningioma

c. Mesotelioma pericardico

d. Mesotelioma della tunica vaginale del testicolo

e. Linfoma non-Hodgkin

6. Quali tra le seguenti sono modalità di esposizione all’amianto? (È possibile barrare più di una risposta)

a. Esposizione domestica, riferita a chi convive con un soggetto esposto professionalmente all’amianto

b. Esposizione ambientale, riferita a chi vive in aree geografiche contaminate da amianto

c. Esposizione occupazionale, riferita a chi svolge un’attività professionale in cui vi sia presenza di amianto

7. Da letteratura qual è il sintomo principale nell’asbestosi utile a indirizzare la diagnosi?

a. Tosse secca

b. Tosse produttiva

c. Dispnea prima da sforzo poi anche a riposo

d. Dolore toracico

e. Dita claviformi

8. In presenza di diagnosi di asbestosi per un lavoratore esposto a rischio di amianto:

a. il medico fa segnalazione online all’INAIL

b. il medico fa segnalazione online a INAIL e INPS

c. il medico fa segnalazione online a INAIL e autorità giudiziaria

d. il medico fa segnalazione online a INAIL, INPS e autorità giudiziaria

e. il medico non ha particolari obblighi in merito

9. Quali sono i segni radiologici dell'asbestosi polmonare? (È possibile barrare più di una risposta)

a. Le placche pleuriche

b. Fini aspetti reticolari basali

c. Un quadro reticolo-nodulare diffuso

d. Broncogramma aereo

e. Aumento degli spazi intercostali

10. Da letteratura qual è la latenza media per il mesotelioma?

a. 2 anni

b. 5 anni

c. 10 anni

d. 15 anni

e. Oltre 25 anni

11. Quale fra le seguenti indagini è più indicata per la diagnosi e la stadiazione del mesotelioma pleurico?

a. Radiografia del torace

b. Tomografia computerizzata

c. Spirometria

d. Scintigrafia

e. Risonanza Magnetica

12. Quale affermazione relativa al mesotelioma della pleura è corretta?

a. L’esposizione all'asbesto è l'unico fattore di rischio

b. Il mesotelioma metastatizza prevalentemente all’apparato osteo-articolare

c. Il fumo di tabacco è causa di mesotelioma solo nei soggetti non esposti all'asbesto

d. L'esposizione all'asbesto e il fumo di tabacco sono fattori di rischio sinergici

e. Tra i diversi istotipi di mesotelioma, il sarcomatoide ha prognosi migliore

13. Da quali delle seguenti strutture anatomiche **NON** origina il mesotelioma? (È possibile barrare più di una risposta)

a. Linfonodi

b. Meningi

c. Pericardio

d. Miocardio

e. Tunica vaginale

14. Secondo la Legge 257/92 “Norme relative alla cessazione dell’impiego dell’amianto”, l’esposizione ad amianto è consentita, con opportuni provvedimenti e misure di protezione del lavoratore, nelle attività di:

a. Estrazione del minerale

b. Produzione di cemento-amianto

c. Coibentazione con amianto

d. Smaltimento e/o bonifica di aree e/o manufatti contenenti amianto

e. Nessuna delle alternative è corretta

***AMBITO DELLA COMPETENZA***

***Le domande seguenti sono finalizzate a rilevare la consapevolezza di competenze specifiche sulle patologie asbesto-correlate.***

***Su una scala da 1 a 5, dove 1 è “Completamente in disaccordo” e 5 è “Completamente d'accordo”, indichi la sua opinione per ognuna delle affermazioni che seguiranno.***

15. Le seguenti azioni rispetto alle malattie professionali rientrano tra le Sue competenze:

- a) Diagnosi 1□□□□□5

- b) Denuncia 1□□□□□5

- c) Compilazione dei certificati medici 1□□□□□5

16. I fattori che rendono difficoltoso denunciare una malattia professionale sono:

- a) Mancanza di conoscenza di procedure burocratiche 1□□□□□5

- b) Difficoltà nell’espletare le procedure burocratiche 1□□□□□5

- c) Mancanza di conoscenza di criteri diagnostici 1□□□□□5

- d) Mancanza di tempo 1□□□□□5

- e) Inadeguato aggiornamento professionale (ECM, etc.) 1□□□□□5

- f) Complessità dell’Elenco delle malattie professionali

per le quali è obbligatoria la denuncia 1□□□□□5

17. “Il Suo attuale livello di aggiornamento scientifico e professionale in tema di malattie asbesto-correlate è adeguato a rispondere in maniera esaustiva alle domande dei suoi assistiti sulle malattie professionali e sugli infortuni nei luoghi di lavoro”.

1□□□□□5

18. “La qualità dell’Educazione Continua in Medicina nella Regione Piemonte sul tema asbesto e patologie correlate è adeguata”.

1□□□□□5

19. “Lei è informato sull’attività lavorativa dei Suoi assistiti”.

1□□□□□5

1. Negli ultimi 12 mesi, ha seguito corsi di Educazione Continua in Medicina che comprendessero anche tematiche relative alle patologie asbesto-correlate?
   1. Sì
   2. No
2. Negli ultimi 12 mesi, Le è capitato di visitare pazienti con patologie correlate all’asbesto?
   1. Sì Se sì, quanti ne ha visitati?____________
   2. No
   3. Non ricordo

1. Negli ultimi 5 anni, quante denunce di malattia professionale ha compilato?

□ 0 □ 1-2 □ 3-4 □ >5

***Le chiediamo ora di completare alcune domande riguardanti i Suoi***

***DATI ANAGRAFICI e PROFESSIONALI:***

1. Età: ______ Genere: F □ M □

Anno di conseguimento della laurea in Medicina e Chirurgia:__________

1. Indichi l’area in cui svolge la Sua professione:
   1. Alessandria
   2. Casale Monferrato
   3. Valenza
   4. Tortona
   5. Novi Ligure
   6. Ovada
   7. Acqui Terme
2. Ha conseguito una o più specializzazioni/corso MMG?
   1. Sì
   2. No

Se Sì, indicare quale/i: _______________________________________________________

__________________________________________________________________________

1. Da quanti anni svolge la Sua professione di medico? (indicare gli anni di attività) ___________
2. Quanti assistiti ha in cura?
   1. Fino a 500
   2. Tra i 501 e 1000
   3. Tra i 1001 e i 1500
   4. Oltre i 1500
3. Considerando sia visite che telefonate, quanti contatti ha giornalmente con i Suoi pazienti?
   1. 0 – 9
   2. 10 – 19
   3. 20 – 29
   4. 30 – 39
   5. 40 – 49
   6. Oltre 50
4. Quanto tempo mediamente è presente nel/i Suo/i ambulatorio/i ogni settimana?
   1. Fino a 5 ore
   2. Tra 5 e 10 ore
   3. Tra 10 e 15 ore
   4. Tra 15 e 20 ore
   5. Oltre 20 ore

Eventuali commenti, osservazioni:

________________________________________________________________________________________________________________________________________________________________________________________________________________________________________________________________________________________________________________________________
